# Identifying a Population of Glial Progenitors That Have Been Mistaken for Neurons in Embryonic Mouse Cortical Culture

**DOI:** 10.1523/ENEURO.0388-20.2020

**Published:** 2021-03-05

**Authors:** Yang Zhang, Beika Zhu, Fulin Ma, Karl Herrup

**Affiliations:** Division of Life Science, Hong Kong University of Science and Technology, Kowloon, Hong Kong

**Keywords:** Alzheimer’s disease, cell cycle, cell death, neurodegeneration, precursor

## Abstract

Experiments in primary culture have helped advance our understanding of the curious phenomenon of cell cycle-related neuronal death. In a differentiated postmitotic cell such as a neuron, aberrant cell cycle reentry is strongly associated with apoptosis. Indeed, in many pathologic conditions, neuronal populations at risk for death are marked by cells engaged in a cell cycle like process. The evidence for this conclusion is typically based on finding MAP2^+^ cells that are also positive for cell cycle-related proteins (e.g., cyclin D) or have incorporated thymidine analogs such as bromodeoxyuridine (BrdU) or 5-ethynyl-2’-deoxyuridine (EdU) into their nuclei. We now report that we and others may have partly been led astray in pursuing this line of work. Morphometric analysis of mouse embryonic cortical cultures reveals that the size of the “cycling” MAP2^+^ cells is significantly smaller than those of normal neurons, and their expression of MAP2 is significantly lower. This led us to ask whether, rather than representing fully developed neurons, they more closely resembled precursor-like cells. In support of this idea, we find that these small MAP2^+^ cells are immunopositive for nestin, a neuronal precursor marker, Olig2, an oligodendrocyte lineage marker, and neural/glial antigen 2 (NG2), an oligodendrocyte precursor marker. Tracking their behavior in culture, we find that they predominantly give rise to GFAP+ astrocytes instead of neurons or oligodendrocytes. These findings argue for a critical reexamination of previous reports of stimuli that lead to neuronal cell cycle-related death in primary cultures.

## Significance Statement

While many laboratories use cultures of rodent embryonic cortex to study the cell biology of brain function, few attend to the identity of the cells in their cultures. We find that a subpopulation of small MAP2-weakly-positive cells, presumed for decades to be neurons, are actually neural/glial antigen 2 (NG2)^+^Olig2^+^Nestin^+^ precursor cells that give rise over time mostly to astrocytes. Subjecting the cultures to a challenge meant to mimic an *in vivo* cell cycle-related neuronal death, we find the percentage of “cycling” MAP2^+^ cells does indeed increase, but only because large MAP2^+^ cells (true neurons) die with no change in their mitotic activity. The MAP2^+^ precursors remain constant in number and in cell cycle activity. This argues for re-interpretation of experimental neuronal culture data.

## Introduction

A substantial number of studies have agreed that re-initiation of a cell cycle process in postmitotic cells such as neurons is tightly associated with apoptosis. This concept has been extended to suggest that cycle-related neuronal death contributes to the pathogenesis of several neurodegenerative disorders including Alzheimer’s disease (AD; Busser et al., 1998; Herrup and Yang, 2007), Parkinson’s disease (Höglinger et al., 2007), ALS (Ranganathan et al., 2001), and others. While the correlations *in vivo* (in humans, mice, and flies) are robust, the detailed molecular mechanisms linking neuronal loss of cycle control to neuronal death are not yet fully understood. In search of these mechanisms, researchers have frequently turned to studies using primary neuronal cultures, primarily of embryonic neocortex or hippocampus. This approach offers a simple and reliable system that allows the manipulation of the cells and their environment. They are usually isolated at late embryonic or early postnatal ages near the end of the neurogenic phase of development. The choice of this early time of harvest is driven by the fact that at this stage, the young postmitotic neurons have not yet developed extensive processes and are relatively resistant to damage during the dissociation protocol (Sciarretta and Minichiello, 2010). While the precise embryonic age chosen for the harvest can impact the identity of the neurons in the culture (Romito-DiGiacomo et al., 2007), most studies involved use embryonic day (E)16.5 mouse embryos or an equivalent gestational age of rat embryos. Culture media have been optimized to specifically favor the long-term survival and growth of fully differentiated neurons over other cell types (Brewer, 1995). Neurons isolated by these procedures remain postmitotic and form functional synapses after maturation making it possible to investigate neuronal development, aging, and death *in vitro* (Lesuisse and Martin, 2002; Dajas-Bailador et al., 2008, 2012; Garwood et al., 2011). Despite this, and even after harvesting on the same gestational day, in the most widely used protocols the resulting preparations contain a diverse and changing set of cell types including astrocytes and oligodendrocyte precursor cells (OPCs) whose percentages change over time in culture (Hui et al., 2016).

Several models based on embryonic cortical culture have been used to investigate cycle-related neuronal death in AD. To generate an *in vitro* model of AD, researchers frequently use the Aβ peptide as a stimulus as it has been reported to induce cell cycle reentry of cultured neurons (Copani et al., 1999; Giovanni et al., 1999; Varvel et al., 2008; Zhang et al., 2008; Bhaskar et al., 2009; Seward et al., 2013; Norambuena et al., 2017). A less-frequently used model is based on Aβ-mediated neuroinflammation. Proinflammatory factors released by Aβ-stimulated microglia have also been used to trigger cell cycle-related death in cultured neurons (Wu et al., 2000; Seward et al., 2013; Bhaskar et al., 2014; Hui et al., 2016; Norambuena et al., 2017; Koseoglu et al., 2019; Zhu et al., 2019).

*In vivo* and under normal conditions *in vitro*, mature neurons never divide. Therefore, a cell that becomes immunopositive for a cell cycle regulatory protein (e.g., a cyclin) is presumed to have lost its cell cycle suppression and re-entered a cell cycle. Viewing this process by immunocytochemistry is superior to techniques such as Western blotting or flow cytometry since it reveals a clear cell-by-cell picture of protein localization and neuron morphology. In addition to cell cycle proteins, bromodeoxyuridine (BrdU)/5-ethynyl-2’-deoxyuridine (EdU) incorporation has also been widely used to detect cell cycle events. To verify that cycling neurons die, BrdU^+^ neurons were double-labeled with the apoptotic markers cleaved caspase-3 or terminal deoxynucleotidyl transferase-mediated biotinylated UTP nick end labeling (TUNEL; Wu et al., 2000). Consistent with the suggestion that the dividing neurons were dying, BrdU+ neurons in culture are smaller in size and lose MAP2^+^ processes (Bhaskar et al., 2009).

In reviewing this rather extensive literature, we became intrigued that photomicrographs that illustrate the cycling neurons consistently showed cells that were considerably smaller than their non-cycling neighbors, with fewer and shorter processes. While this seemed reasonable, as the cycling neurons would be expected to be unhealthy (Varvel et al., 2008; Bhaskar et al., 2009, 2014; Hui et al., 2016; Norambuena et al., 2017), several findings in our lab led us to question this assumption. We realized that no study, including our own, provided evidence that these small cycling neurons came from normal healthy neurons.

We report here that the “dividing” MAP2^+^ cells are not actual mature cortical neurons. Though positive for MAP2, they are also immunopositive for nestin, Olig2, and neural/glial antigen 2 (NG2). They thus are more accurately seen as progenitor cells. We find that with time, rather than dying, they differentiate into astrocytes. Our data emphasize the importance of re-examining and more carefully defining the cell types present in primary cortical culture and suggest that many of the conclusions reached in previous *in vitro* studies of neuronal cell cycle activity may have to be re-examined.

## Materials and Methods

### Animals and primary cortical neuronal culture

The C57BL/6 mice were maintained and bred in in the Animal and Plant Care Facility of The Hong Kong University of Science and Technology (HKUST). Primary cortical cultures were performed following standard protocols as described (Hui et al., 2016). Cervical dislocation was performed to kill the pregnant female C57BL/6 mice, followed by isolation of embryonic mice. Isolated cerebral cortices from E16.5 or P5 C57BL/6 mice were dissociated into single cells by digestion with 0.25% trypsin-EDTA (Thermo Fisher Scientific) and gentle trituration. After neutralization with DMEM containing 10% fetal bovine serum (FBS; Thermo Fisher Scientific), cells were suspended in Neurobasal medium supplemented with B27 and 2 mm GlutaMAX (Thermo Fisher Scientific), and then seeded on glass coverslips or dishes coated with poly-L-lysine (Sigma). Half of the medium was replaced with fresh every 5 d.

### THP-1 cell culture and conditioned medium

Human THP-1 monocytes were grown in RPMI 1640 medium (Thermo Fisher Scientific) supplemented with 10% FBS and 0.05 mm β-mercaptoethanol (Sigma) at 37°C in a humidified 5% CO_2_ atmosphere. The conditioned medium was prepared as described previously (Zhu et al., 2019). To stimulate the THP-1 cells, 2.27 μl of 220 μm fibrillar Aβ_1–42_ (rPeptide) was added to the surface of each well of a 24-well plate and allowed to air dry. THP-1 cells were plated on the Aβ-coated well surface at a density of 100,000 cells per well and cultured in Neurobasal medium for 3 d. The medium was removed, and after centrifugation, the supernatant (5%, v/v) was used to treat neuronal cultures for 24 h.

### Histology, cell fixation, and immunofluorescence

Postnatal day (P)5 C57BL/6 mouse pups were perfused with ice-cold PBS. After perfusion, the brains were dissected and immersed in 4% paraformaldehyde (PFA; Sigma) at 4°C overnight. The fixed brains were cryoprotected by incubating in PBS containing 30% sucrose at 4°C overnight, followed by embedding in OCT. After cryostat sectioning at 10 μm, samples were either used immediately for immunohistochemistry or stored at −80°C. Before immunofluorescence, sections were heated to 95°C in citrate buffer (pH 6.0) for 10 min for antigen retrieval. To prepare them for immunostaining, cortical neuron cultures were first washed in PBS, then fixed in ice-cold 4% PFA for 20 min.

To immunostain, cells or brain sections were blocked with 5% donkey serum in PBS containing 0.3% Triton X-100 at room temperature (RT) for 1 h and then incubated with primary antibodies in blocking solution at 4°C overnight. After rinsing with PBS, samples were incubated with fluorophore-conjugated secondary antibodies at RT for 1 h, followed by three washes in PBS and counter-staining with DAPI for 5 min. After rinsing, samples were mounted with Hydromount media (National diagnostics). Images were taken using Leica TCS SP8 confocal laser scanning microscope (63×, Z-stack specifically for IHC of P5 mouse brain sections) or Olympus DP80 fluorescent microscope (20×). For IHC of P5 mouse brain sections, the gray matter of cortex was imaged.

### EdU-incorporation assay

EdU was used to monitor DNA synthesis in the cultured cortical neurons; 5 μm EdU was added to the cell culture for 6 h before fixation with 4% PFA. EdU labeling was performed according to the manufacturer’s protocol using the Click-iT EdU assay kit (Thermo Fisher Scientific). After EdU labeling, the samples were processed for immunofluorescence or DAPI labeling as described above.

### Measurement of sizes of the MAP2^+^ cells

To measure cell size, neurons were stained with MAP2, and the boundaries of the labeled cells were marked using the free-hand selection tool of the Fiji software package. The selected region area was tabulated separately for MAP2^+^ cells that were positive or negative for cell cycle markers. Size-frequency histograms were then assembled with bin sizes of 20 μm^2^.

### Antibodies

Prmary antibodies against Ki67 (#ab15580, RRID: AB_443209), cyclin D1 (#ab16663, RRID: AB_443423), cyclin A2 (#ab181591), PCNA (#ab29, RRID: AB_303394), MAP2 (#ab5392, RRID: AB_2138153), Nestin (#ab6142, RRID: AB_305313), GFAP (#ab7260, RRID: AB_305808), and β-III tubulin (#ab78078, RRID: AB_2256751) were purchased from Abcam. Mouse anti-GFAP (#G3893, RRID:AB_477010) antibody was purchased from Sigma. Olig2 (#AB9610, RRID: AB_570666) and NG2 (#AB5320, RRID: AB_91789) antibodies were purchased from Millipore. Alexa Fluor 488 or 647-conjugated secondary antibodies were purchased from Thermo Fisher Scientific. Anti-chicken Cy3 antibody was purchased from Jackson ImmunoResearch.

### Statistical analysis

Statistical analysis was performed using Prism 6 (GraphPad software) with further details provided in Results and statistical table in Table 1. Data from at least three independent experiments were presented as mean ± SEM. Differences between each group were assessed with unpaired Student’s *t* test or one-way ANOVA; *p* < 0.05 was considered statistically different; *p < 0.05, **p < 0.01, ***p < 0.001, ****p < 0.0001.

**Table 1:** Statistical tests used in this study

Figure	Data structure	Type of test	Power
1*L*	Unknown	Unpaired *t* test, two-tailed	(1) Ki67, cycling neurons vs non-cycling neurons: *p* < 0.01; 95% confidence interval 28.08, 113.0 (2) Cyclin D1, cycling neurons vs non-cycling neurons: *p* > 0.05; 95% confidence interval −7.889, 67.35 (3) Cyclin A2, cycling neurons vs non-cycling neurons: *p* < 0.0001; 95% confidence interval 69.58, 91.78 (4) PCNA, cycling neurons vs non-cycling neurons: *p* < 0.05; 95% confidence interval 23.03, 110.6 (5) EdU, cycling neurons vs non-cycling neurons: *p* < 0.0001; 95% confidence interval 65.82, 85.25
2*C*	Unknown	Unpaired *t* test, two-tailed	(1) Total, control vs 5% AM: *p* < 0.0001; 95% confidence interval −187.9, −116.6 (2) Big neurons, control vs 5%AM: *p* < 0.0001; 95% confidence interval −175.5, −114.0 (3) Small neurons: control vs 5%AM: *p* = 0.4469; 95% confidence interval −30.05, 15.05
2*D*	Unknown	Unpaired *t* test, two-tailed	(1) Total, control vs 5% AM: *p* = 0.0052; 95% confidence interval 0.03192, 0.1175 (2) Small neurons: control vs 5%AM: *p* = 0.5184; 95% confidence interval −0.08946, 0.1591
5*F*	Unknown	One-way ANOVA	(1) DIV3 vs DIV10: *p* = 0.0235; 95% confidence interval −0.3514, −0.01942 (2) DIV3 vs DIV14: *p* < 0.0001; 95% confidence interval −0.5301, −0.2091
5*G*	Unknown	One-way ANOVA	(1) DIV3 vs DIV10: *p* = 0.0005; 95% confidence interval −0.3214, −0.08521 (2) DIV3 vs DIV14: *p* < 0.0001; 95% confidence interval −0.3963, −0.1690

## Results

### Cycling MAP2^+^ cells are distinct from neurons

**Figure 1.**
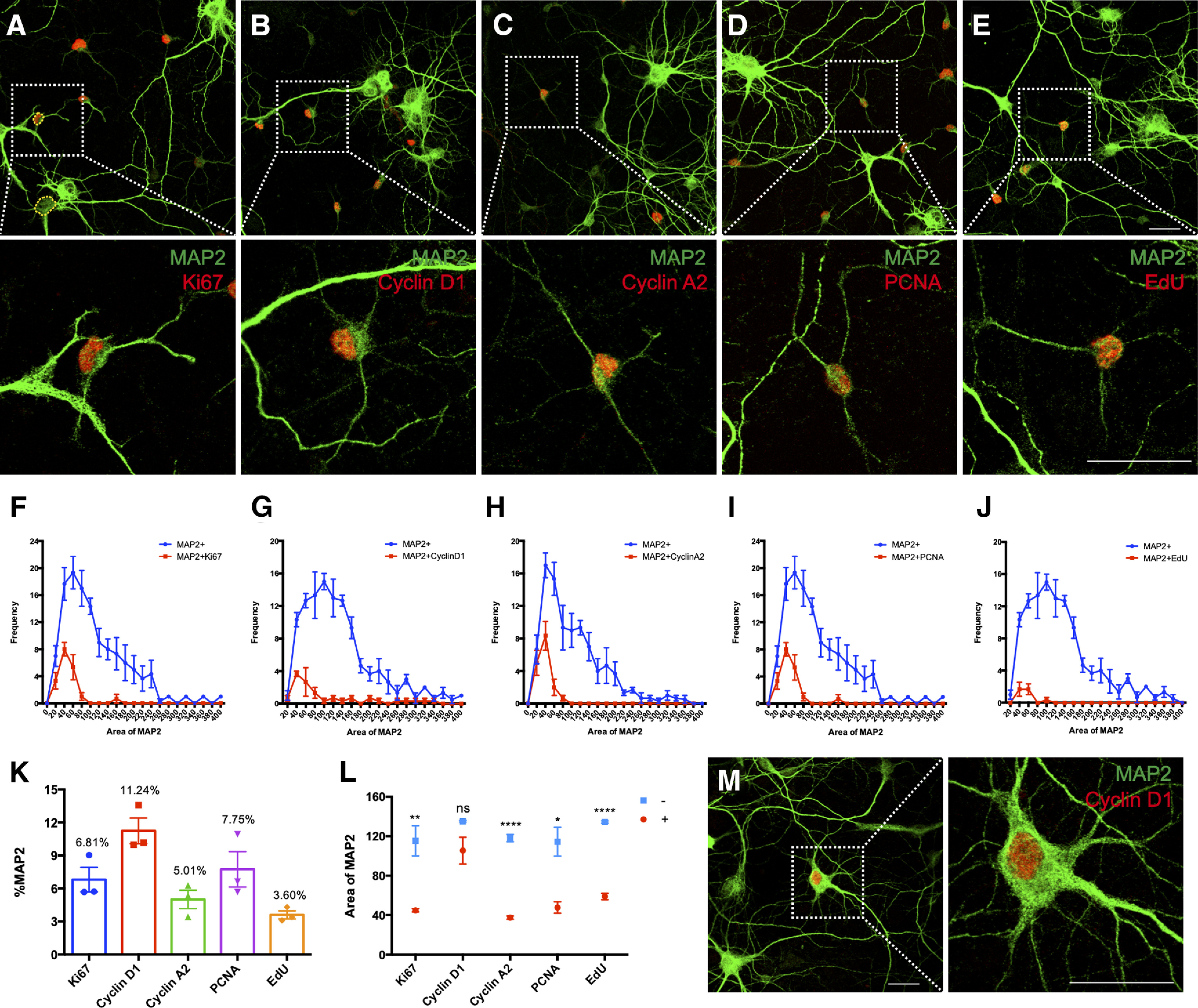
Detection of cell cycle markers in E16.5 mouse primary cortical culture. ***A–D***, Immunostaining for the indicated cell cycle regulatory proteins in DIV14 cortical culture. ***E***, EdU incorporation assay was used to detect the cells that synthesized DNA. ***F–J***, Cell body areas of MAP2^+^ cells were measured using Fiji software. Hand-traced measurement of cell body was conducted as illustrated by the yellow dashed line shown in ***A***. Size-frequency histograms were constructed to compare the relative sizes of marker-positive and negative cells. More than 80 MAP2^+^ cells were quantified on each coverslip (*N* = 3). ***K***, MAP2^+^/cell cycle marker double-positive cells expressed as a percentage of total MAP2^+^ cells. ***L***, Average cell body areas of MAP2^+^ cycling cells (red) and their non-cycling counterparts (blue). ***M***, Morphology of a big neuron expressing nuclear cyclin D1. Data from three batches of culture were presented as mean ± SEM. Scale bars: 25 μm. ns: not significant; *p < 0.05; **p < 0.01; ****p < 0.0001.

Embryonic cortical neurons in culture have been widely used to investigate the cell cycle events that accompany neuron death in a variety of neurodegenerative diseases. Most of these studies rely on the appearance and disappearance of different cell cycle proteins such as cyclin D1 as a marker for the G_1_ phase of the cell cycle, cyclin A2 and PCNA for S phase, and cyclin B1 for G_2_/M phase. Some studies also use the Ki67 protein as a marker for cells in any stage of the cell cycle. Thus, we and others have reported numerous examples of cell cycle activity in primary mouse cortical neurons using immunocytochemistry (Fig. 1*A–D*). As observed previously, when we quantify resting untreated cortical cultures after DIV14 (14 days in vitro), we found a small but significant background of “cycling” neurons ranging from 5% to 10% of the total, regardless of the protein marker used (Fig. 1*K*). To look specifically at DNA replication, we used EdU incorporation and found that ∼3% of the MAP2^+^ cells in our culture synthesized DNA (Fig. 1*E*,*K*).

**Figure 2.**
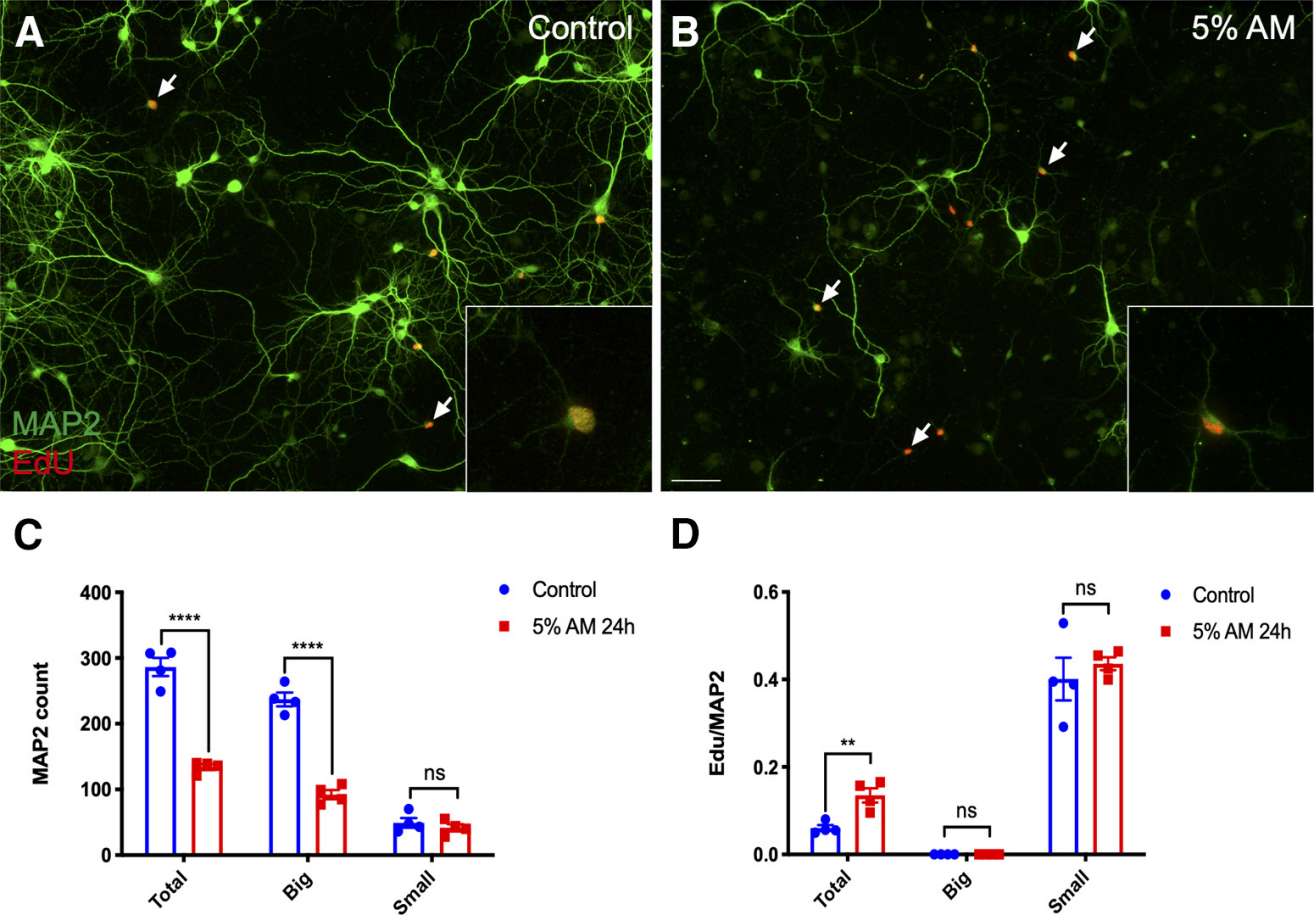
AM treatment does not influence the cell cycle or fate of small neurons. DIV14 cortical cultures from E16.5 mice were treated with 5% AM for 24 h. EdU labeling was used to detect cells with active DNA synthesis. ***A***, ***B***, Representative fields (20×) showing small neurons incorporating EdU (inset is a higher magnification of a single cell from the field). ***C***, Quantification of the number of MAP2^+^ cells, separated by size, after AM treatment. ***D***, Quantification of the ratio of double-positive EdU^+^/MAP2^+^ cells to total MAP2^+^ cells remaining after AM treatment. Data from three independent experiments, presented as mean ± SEM. Scale bars: 50 μm. ns: not significant; **p < 0.01; ****p < 0.0001.

While we have repeated observations such as these many times, we became troubled that the cycling neurons were all relatively small compared with most of the other MAP2^+^ cells in our cultures. We had always assumed that their small size and weaker MAP2 staining intensity were because their unscheduled cell cycle event caused them to shrink and enter an unhealthy state. As we had never done anything to test this impression, we measured the area of the cell body of all MAP2^+^ cells and plotted the cell cycle positive and negative cells separately (Fig. 1*F–J*). We found that, regardless of the cell cycle marker used (except cyclin D1), the average cell body size of the cycling neurons was significantly smaller than the size of their non-cycling counterparts (Fig. 1*L*), suggesting that they are in a very different biological state. We had seen this result before. Inspecting the photographs from earlier publications (Hui et al., 2016) shows that the EdU-positive neurons in experiments such as these were small in size with weak MAP2 staining just like the cells shown in Figure 2*A*,*B*.

There was one exception to the atrophic appearance of the cycling neurons. We consistently found a subpopulation of the neurons labeled with cyclin D that was larger and more normal in appearance. While ∼60% of the MAP2/cyclin D-positive cells were small in size (≤100 μm^2^), the remainder had cell sizes more similar to non-cycling cells (Fig. 1*G*). The morphology of the processes of the two sizes of cyclin D1 positive neurons was noticeably different as well (Fig. 1*D*,*M*). The bigger cells had healthy, large caliber, MAP2^+^ dendrites. One possible explanation of this finding is that since cyclin D is the first marker to appear in a normal cell cycle, perhaps we had caught these cells just as they entered a pathologic cell cycle.

To test this, we challenged our cells with an Alzheimer’s like environment to trigger cell cycle-related neuronal deaths. Our lab has previously used such an *in vitro* model system to investigate the contribution of inflammation to the pathogenesis of neurodegenerative disease. The paradigm involves treating microglia or THP-1 monocytes with β-amyloid, then using the conditioned medium from these cultures to treat primary neurons in culture (Wu et al., 2000; Hui et al., 2016). Typically, this amyloid-stimulated conditioned medium (AM) increases the percentage of EdU-positive neurons from ∼5% to ∼15% (Hui et al., 2016). We repeated this experiment for this study and obtained similar results (Fig. 2). Cell counts revealed that after 24 h of AM treatment, over 60% of the total MAP2^+^ cells had been lost and, of the survivors, the percentage of MAP2^+^ cells that was labeled with EdU increased more than two-fold (Fig. 2*C*,*D*).

While these results are consistent with earlier studies, a different picture emerged when we examined small and big neurons separately. After 24 h of AM treatment, over 60% of the population of large neurons was lost. Despite this, not a single EdU-positive neuron was observed in this group (Fig. 2*C*,*D*). By contrast, the small neurons suffered virtually no cell loss, and what is more, the percentage of small neurons that was EdU-positive was not significantly changed (Fig. 2*C*,*D*; statistical table is provided in Table 1). Therefore, the increase of the ratio of cycling to total MAP2^+^ cells after AM treatment was actually caused by the death of the large neurons not by an increase in cell cycle activity. While unexpected, these findings lead to the conclusion that that AM has no effect on either the cell cycle activity or the fate of the small neurons in our cultures and raises questions concerning their identity.

### The “small neurons” have characteristics of neuronal progenitors

**Figure 3.**
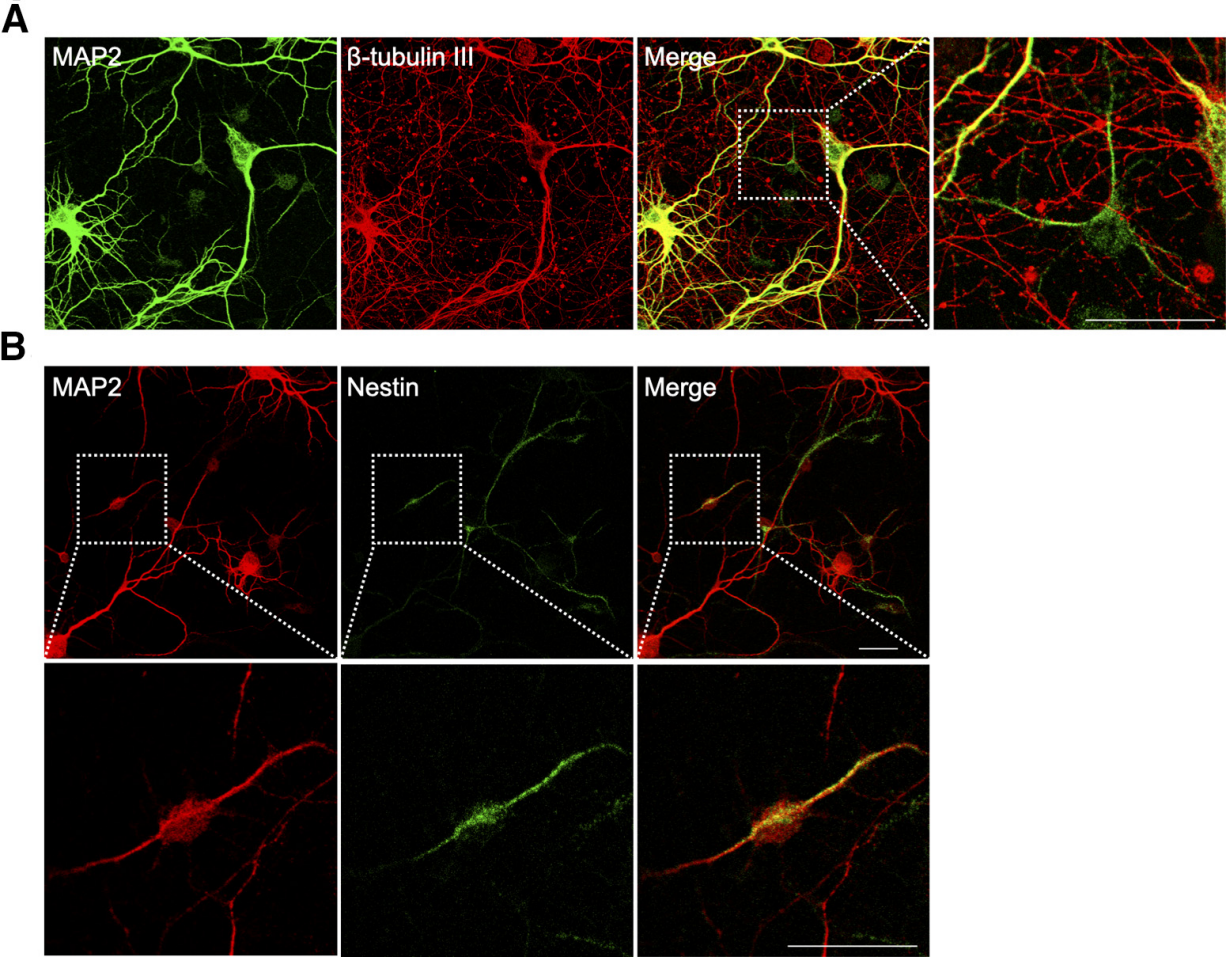
The small neurons in E16.5 mouse cortical culture express the neural progenitor marker nestin. ***A***, DIV14 culture was immunostained with antibodies against neuronal markers MAP2 and β-III tubulin. ***B***, Immunostaining of nestin in DIV14 cortical culture. A representative cell expressing nestin and MAP2 is shown enlarged in the bottom panels. Scale bars: 25 μm.

Since the small neurons were mitotically active cells, we asked whether they had any attributes that might mark them as neural progenitors. To do this, we stained our cultures with antibodies against nestin, an intermediate filament protein that is expressed in neural stem/progenitor cells found in the ventricular zone of the developing brain (Zimmerman et al., 1994). We found that virtually all of the small neurons were nestin^+^ (Fig. 3*B*). During a normal developmental progression, when nestin^+^ precursor cells in the ventricular zone exit the cell cycle and begin the process of migration, they stop producing nestin. During their migration and early maturation, the young postmitotic neurons begin expressing β-III tubulin, an embryonic isomer of adult β-tubulin (Menezes and Luskin, 1994; Sullivan, 1988). With further maturation β-III tubulin also disappears and adult cells end up expressing exclusively MAP2, a microtubule-associated protein found in mature dendrites. Finding nestin in MAP2^+^ cells is unexpected since these two markers are normally expressed at very different developmental stages. To further define the developmental identity of the small neurons, we stained our cultures with β-III tubulin. We found that the small MAP2^+^ neurons were β-III tubulin-negative, whereas all big neurons were strongly labeled (Fig. 3*A*). Finding β-III tubulin co-localized with MAP2^+^ neurons was unexpected, as it suggests that despite their morphology, the biochemistry of the cultured neurons has not fully matured. The absence of β-III tubulin in the dual presence of nestin and MAP2 can be taken as evidence that that the small neurons are much more like precursor cells and thus presumably not real neurons.

### The small neurons express NG2 but do not develop into oligodendrocytes

**Figure 4.**
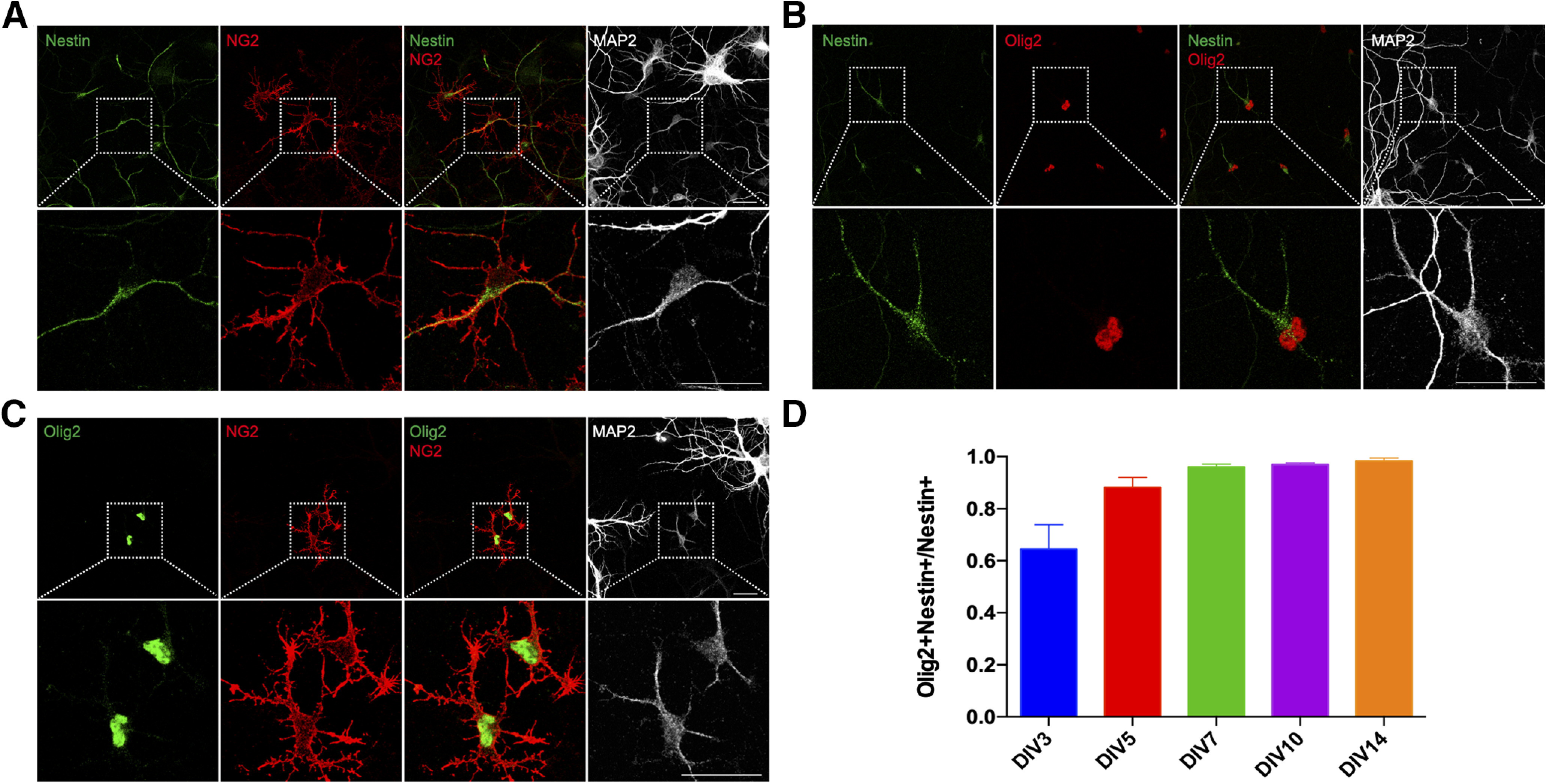
The small neurons express Olig2 and NG2 but do not develop into oligodendrocytes in culture. DIV14 cortical cultures from E16.5 mice were double labeled with antibodies against (***A***) nestin and NG2, (***B***) nestin and Olig2, (***C***) Olig2 and NG2. ***D***, Percentage of the number of nestin^+^/Olig2^+^ double-positive cells expressed as a fraction of total nestin^+^ cells. Scale bars: 25 μm.

Neural progenitors have been shown to be able to differentiate into diverse cell types, including neurons, astrocytes, or oligodendrocytes (Mignone et al., 2004). While the small neurons express MAP2, it is unlikely that they are capable of developing into cortical neurons as they expressed neither β-III tubulin nor Tbr2, a marker of intermediate neuronal progenitor cells (Englund et al., 2005; data not shown). In testing whether they might be oligodendrocyte progenitors, we found that the small neurons were immunopositive for NG2 (Fig. 4*A*), a proteoglycan expressed specifically in OPCs or NG2-glia of the brain (Richardson et al., 2011). We, therefore, speculated that the small neurons were in reality cells of the oligodendrocyte lineage cells. We verified this possibility by immunostaining cultures with the oligodendrocytes lineage marker Olig2 (Fig. 4*B*,*C*; Zhou et al., 2000). While the presence of these two proteins would tend to identify the small neurons as OPCs, there was no sign of their further differentiation in our cultures. Thus, no cell in our culture was found to express the mature oligodendrocyte marker, myelin basic protein (MBP; data not shown).

### The small neurons generate astrocytes

**Figure 5.**
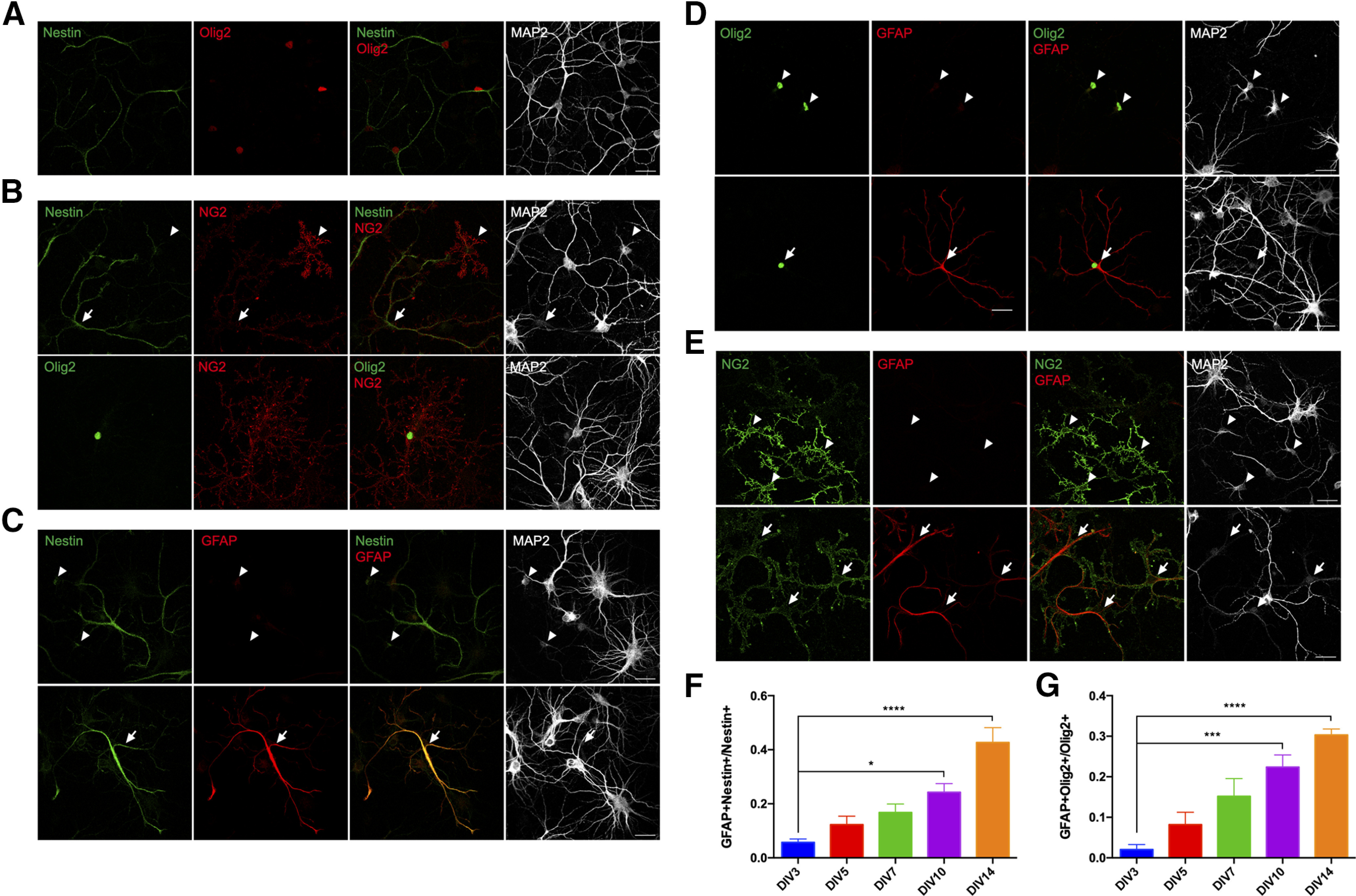
The small neurons generate astrocytes in E16.5 mouse cortical culture. ***A***, Immunostaining reveals nestin^+^Olig2^+^ cells in addition to the small neurons in DIV14 culture. ***B***, Double labeling of nestin (upper panels) or Olig2 (lower panels) and NG2 reveals large nestin-negative cells with weaker and diffuse NG2 signal (arrow) compared with that of the small neurons (arrowhead). ***C***, Double labeling of nestin and GFAP in DIV14 culture reveals big nestin cells expressing GFAP (arrow), while the nestin^+^ small neurons are GFAP-negative (arrowheads). ***D***, Double labeling of Olig2 and GFAP in DIV14 culture reveals big Olig2^+^ cells expressing GFAP (arrow); nestin^+^ small neurons do not express GFAP (arrowheads). ***E***, Double labeling of NG2 and GFAP in DIV14 culture shows NG2 cells with weak and diffuse NG2 signals expressing GFAP (arrows); the NG2^+^ small neurons do not express GFAP (arrowheads). ***F***, ***G***, Ratio of GFAP/nestin double-positive cells to total nestin^+^ (***F***) or GFAP/Olig2 double-positive cells to total Olig2^+^ cells (***G***) gradually increases from DIV3 to DIV14. Data from at least three independent cultures are presented as mean ± SEM. Scale bars: 25 μm. *p < 0.05; ***p < 0.001; ****p < 0.0001.

It seemed that the small neurons could not generate either neurons or oligodendrocytes; we therefore asked whether they were able to differentiate into astrocytes. In our cultures, we observed a number of nestin^+^ cells with a morphology that was distinct from that of the small neurons. These nestin^+^ cells were larger in size with more processes than were found on the small neurons. While they looked fully differentiated, they showed less MAP2 expression in processes (Fig. 5*B*). In DIV14 cultures, over 95% of the nestin^+^ cells, including the small neurons, expressed nuclear Olig2 (Fig. 4*B*,*D*). By contrast, these big nestin^+^ cells (also Olig2^+^) exhibited a weaker, diffuse NG2 signal. That raised the possibility that these larger cells were derived from small neurons and that the expression of NG2 declined during their transformation (Fig. 5*B*, arrow). In keeping with this idea, we found that a large proportion of the big nestin^+^ cells expressed the astrocyte marker GFAP, co-localized with the nestin immunoreactivity (Fig. 5*C*, lower panel). Nestin^+^Olig2^+^ small neurons did not express GFAP (Fig. 5*C*,*D*, upper panel), but many Olig2^+^ cells expressed GFAP as well (Fig. 5*D*, lower panel). From DIV3 to DIV14, the percentage of nestin^+^ cells that were GFAP^+^ gradually increased (Fig. 5*F*). During the same time period, the percentage of Olig2^+^ cells that were GFAP^+^ also significantly increased (Fig. 5*G*). Thus, almost all of the GFAP^+^ cells in the culture were nestin^+^Olig2^+^. NG2 immunostaining could be found in the large GFAP^+^ nestin^+^ cells, but it was always weak and diffuse (Fig. 5*E*, lower panel). This decline in NG2 staining distinguishes the GFAP^+^ cells from the small neurons where no GFAP expression could be detected despite the presence of NG2 (Fig. 5*E*, upper panel). Taken together, our data suggest that MAP2^+^/nestin^+^/Olig2^+^/NG2^+^ small neurons differentiate into GFAP^+^ astrocytes in the culture.

### The small neurons in mouse brain also give rise to astrocytes

**Figure 6.**
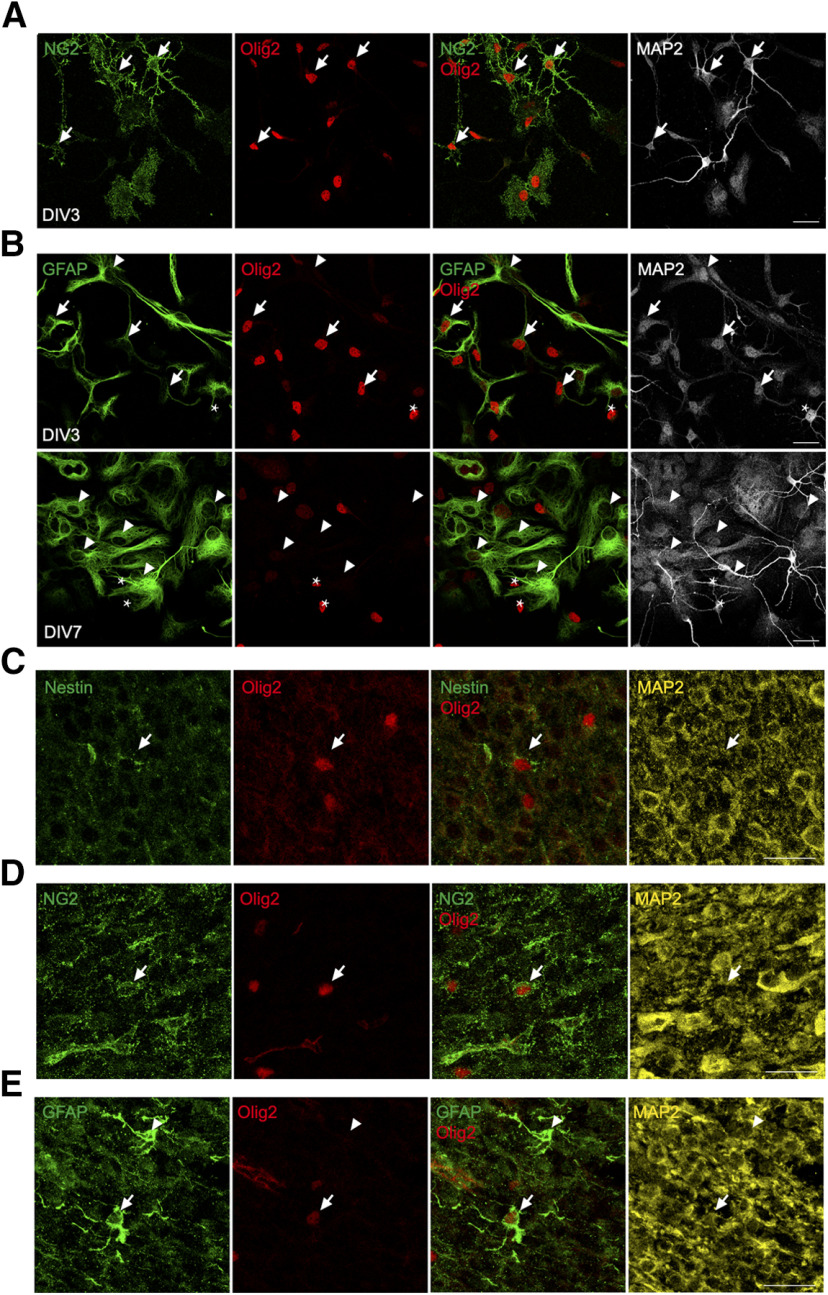
Detection of small neurons in cortical cultures from P5 mice and in P5 mouse brain. ***A***, Immunostaining of Olig2, NG2, and MAP2 reveals the presence of small neurons (arrows) in DIV3 cortical culture from P5 mouse. ***B***, Double labeling of Olig2 and GFAP shows astrocytes expressing Olig2 (arrows) or not (arrowheads) in DIV3 (upper panel) or DIV7 (upper panel) cortical culture from P5 mouse. Stars denote the small neurons. ***C***, Immunostaining of an Olig2^+^nestin^+^ cell (arrow) in the cortex of a P5 mouse. ***D***, Immunostaining of an NG2^+^Olig2^+^ cell (arrow) in the dorsal (motor) cortex of a P5 mouse. ***E***, Double labeling of Olig2 and GFAP showing astrocytes expressing Olig2 (arrows) or not (arrowheads) in the cortex of P5 mice. Scale bars: 25 μm.

It would appear that the cycling small neurons in cultures of E16.5 mouse cortical are progenitor cells that differentiate into astrocytes. However, the question arises: are such cells an artifact of culture, or can they also be found *in vivo*? In E16.5 cultures, GFAP^+^ astrocytes first appear around DIV5, after which their number gradually increases up to at least DIV14 (Fig. 5*F*,*G*). We isolated cortex from P5 mice and established dissociated cultures using the same protocol for E16.5 cortex. In the P5 cortical culture, after 3 d, many characteristic Olig2^+^NG2^+^ small neurons began to emerge (Fig. 6*A*) along with many GFAP^+^ astrocytes (many of which were also Olig2^+^; Fig. 6*B*, upper panel). This is much faster than such cells appeared in E16.5 cultures. During differentiation, as expected, these GFAP^+^ cells gradually lost their Olig2 (Fig. 6*B*, lower panel). That is to say, in P5 culture the emergence of Olig2^+^NG2^+^ small neurons and Olig2^+^GFAP^+^ astrocytes occurred more quickly than in E16.5 culture. This suggests that the small neurons are still present in the P5 brain and that they have begun to differentiate *in vivo* allowing them to establish themselves faster in dissociated culture. To further confirm this, we directly performed immunohistochemistry of the P5 mouse brain. Immunostaining of developing neocortex revealed a complex cellular landscape. Nonetheless, we were able to detect a small number of Olig2^+^nestin^+^ (Fig. 6*C*) and Olig2^+^NG2^+^ (Fig. 6*D*) small neurons in the cortex of P5 mice as predicted from the *in vitro* work. We also observed GFAP^+^/Olig2^+^ (Fig. 6*E*) cells, suggesting Olig2^+^ cells also differentiate into GFAP^+^ astrocytes in mouse brain. These cells were relatively rare and scattered throughout the cortex with no obvious regional variability. These data are consistent with the idea that a population of cells very similar to the small neurons we identified *in vitro* exist in the early postnatal mouse brain and further that these precursor-like cells develop into astrocytes *in vivo*.

## Discussion

Ectopic neuronal cell cycle reentry is tightly associated with neuronal cell death and is often cited as an integral part of the pathogenesis of neurodegenerative disorders such as AD (Park et al., 1997; Busser et al., 1998; Copani et al., 1999; Kruman et al., 2004; Herrup and Yang, 2007; Folch et al., 2012; Seward et al., 2013; Norambuena et al., 2017). Most of the evidence supporting this disease model has come from descriptive correlations observed in neuropathological samples. Few if any controlled experimental manipulations have been done *in vivo* as such experiments are difficult to perform. For this reason, virtually all of the studies aimed at understanding the molecular mechanisms of cycle-related neuronal death have been done *in vitro*, in dissociated cultures of embryonic neurons. As with their *in vivo* counterparts, these cultured neurons exit the cell cycle and generate electrically active processes. The advantage of this preparation is that, in the controlled environment of a tissue culture dish, a variety of stimuli such as DNA damage (Park et al., 2000; Kruman et al., 2004; Liu et al., 2014), oxidative stress (Yang et al., 2014), proinflammatory cytokines (Varvel et al., 2009; Bhaskar et al., 2014) and other manipulations can and have been used to induce ectopic neuronal cell cycle events. For many labs including our own, the outcome measure used to monitor this process is the appearance of cells that are double-immunolabeled with MAP2 and a cell cycle protein (or BrdU/EdU incorporation). The assumption has always been that these double-labeled cells were mitotically active neurons.

Our current study challenges this assumption. We performed double-labeling of cultured neurons using antibodies against MAP2 and a variety of cell cycle proteins, including Ki67, cyclin D1, cyclin A2, and PCNA. We first showed that the double-labeled cells formed a distinct cell population that was small in size (<100 μm^2^) with thin dendritic branches and significantly weaker MAP2 immunolabelling. Consistent with the cell cycle protein staining, EdU incorporation was only found in these cells. Building on this observation, we used cell body size to divide our cultures into two groups for analysis. We challenged our cultures with conditioned medium from THP-1 cells stimulated with Aβ. As expected, this manipulation increased the total percentage of EdU/MAP2 double-labeled cells. The effects of conditioned medium, however, were drastically different in the two different size classes of neurons. Larger (normal looking) neurons were killed by the conditioned medium, but never incorporated EdU. The smaller MAP2^+^ cells continued to incorporate EdU but showed no response to AM, their numbers did not change, nor did the percentage that were EdU labeled.

These observations have direct implications for previous cell cycle/cell death studies in neurons. Figure 2*C*,*D* repeats findings that have been replicated by many authors, including our lab. The interpretation of this simple experiment is that after AM, neuronal cell cycling increases, and when it does, the cycling neurons die. This may yet be true *in vivo*, but *in vitro* Figure 2*E–H* tells a cautionary tale. Large, fully mature MAP2^+^ neurons die in response to microglial conditioned medium, but they do not enter a cell cycle before doing so. The MAP2^+^ cells we have called small neurons remain as the only mitotic cells in culture. When, after AM, the ratio of cycling to total MAP2^+^ cells increases in culture, the increase is caused entirely by the death of the large neurons (the denominator decreases) not by an increase in cell cycle activity (the numerator remains unchanged; Fig. 2*H*). The inescapable conclusion is that many studies in this field need to be re-examined and perhaps reinterpreted.

The cells we called small neurons are not real neurons although they have processes and express MAP2. Instead, we find that they are nestin^+^/Olig2^+^/NG2^+^ progenitor cells. They cannot be committed to a neuronal lineage, since we seldom detect cells that are double positive for β-III tubulin and nestin (or Olig2). Although they do express Olig2 and NG2, markers associated with the oligodendrocyte lineage, the absence of mature oligodendrocytes suggests that they do not differentiate into oligodendrocytes, at least in our culture conditions. Their most likely fate can be found in our observation of GFAP^+^ astrocytes that also express nestin and Olig2. While the co-expression of MAP2, Olig2, GFAP (neuron, oligodendrocyte, astrocyte proteins) along with proteins such as nestin that are found in immature ventricular zone precursor cells suggests that there are strong molecular cross-currents buffeting the fate decision of these cells, our data suggest that these unusual cells most probably give rise to astrocytes in our culture system with time. To begin, we find cells expressing both GFAP and NG2, and it has been reported that NG2 glia generate astrocytes in addition to oligodendrocytes (Zhu et al., 2008). We and others have also found that the number of astrocytes increases steadily with time. The nestin^+^/Olig2^+^/NG2^+^ small neurons thus serve as a precursor pool for an ever-enlarging population of astrocytes.

While the field has historically referred to the cycling MAP2^+^ cells in mouse embryonic cortical culture as neurons, the data show that they are instead glial progenitors that express NG2, MAP2, Olig2, and nestin. During their differentiation, these progenitors grow thicker processes and grow larger in size. At the same time, the MAP2 and NG2 proteins diffuse into their cytoplasmic processes and gradually degrade. During this same differentiation period, GFAP expression is established, and Olig2 and nestin are ultimately degraded (Olig2 preceding nestin). The expression of nestin and Olig2 in astrocytes finds precedent in the literature. While its exact function has yet to be determined, developing astrocytes in mouse brain are known to express Olig2 as they become specified and differentiated, gradually reducing expression as maturation is completed (Marshall et al., 2005). Nestin is a well-known component of radial glial cells – GFAP^+^ astroglial cells in developing mouse brain, so the co-expression of these two proteins also has precedence (Wei et al., 2002). Nestin is not expressed in mature astrocytes, but after injury, re-expression of nestin was observed in reactive astrocytes (Schmidt-Kastner and Humpel, 2002). The constant co-expression of nestin and GFAP in astrocytes in our culture suggests astrocytes in embryonic cortical culture are not fully mature, probably because of lack of astrocyte maturation factors. The existence of Olig2^+^/NG2^+^ and nestin^+^/NG2^+^ cells in the P5 mouse brain and cortical cultures established from P5 mouse indicates that the small neurons are not artifacts of culture. GFAP^+^ astrocytes expressing weak Olig2 in the nucleus confirms Olig2^+^ cells also develop into astrocytes in the brain of postnatal mice.

**Figure 7.**
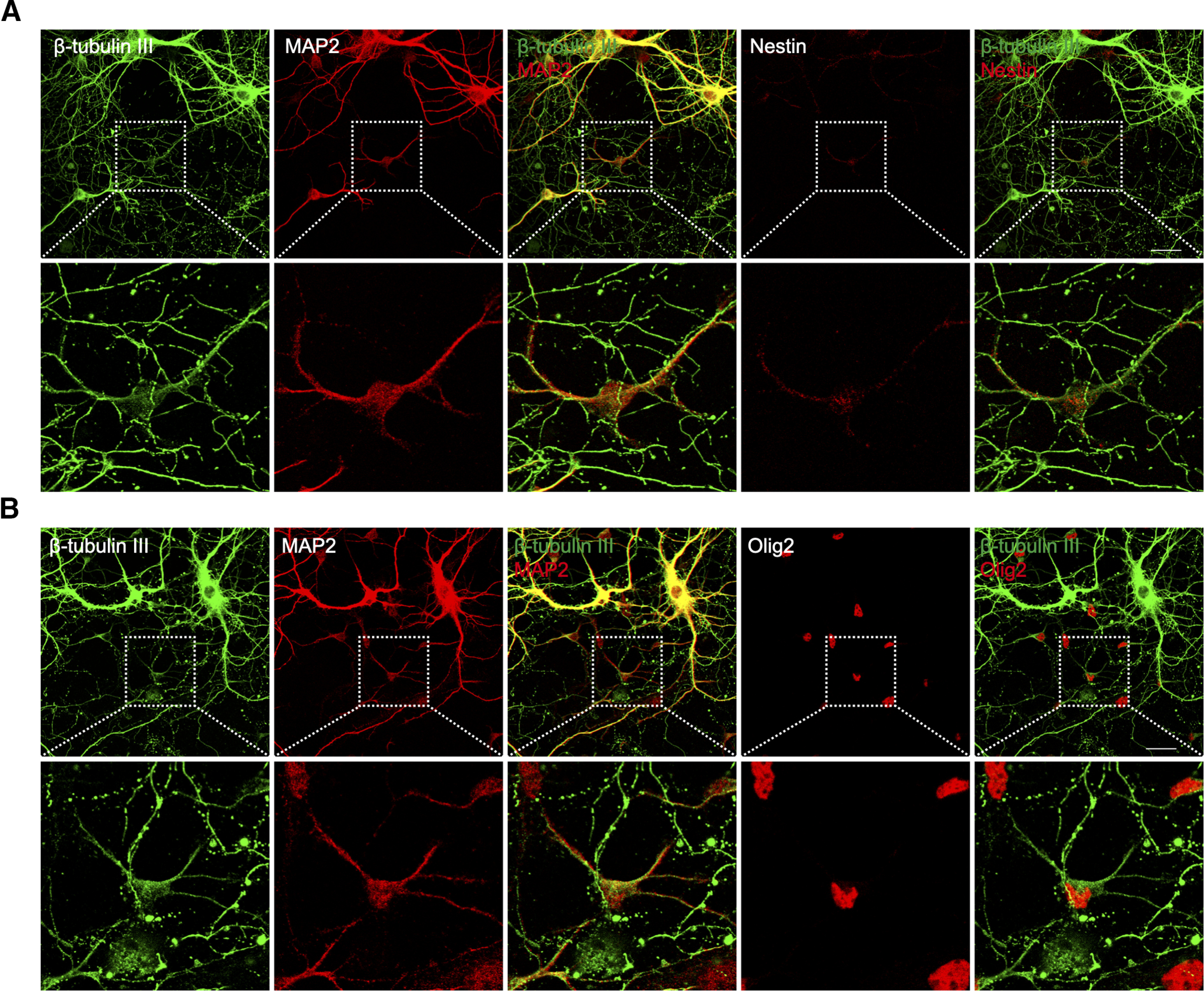
Expression of β-III tubulin by some small neurons in DIV21 cortical culture from E16.5 mice. ***A***, Immunostaining showing nestin^+^ small neurons expressing β-III tubulin. ***B***, Immunostaining showing Olig2^+^ small neurons expressing β-III tubulin. Scale bars: 25 μm.

Confusion in the field has arisen largely because these small neurons/astroglial progenitors also express MAP2 in soma and processes. Traditionally, MAP2 is a neuron-specific cytoskeletal protein enriched in dendrites where it helps to stabilize microtubules. Like tau, however, of MAP2 has several alternatively spliced isoforms (Kalcheva et al., 1995). One of these includes exon 13 (MAP2 + 13) and has been found to be transiently expressed in oligodendrocyte/astrocyte precursor cells during human CNS development (Blümcke et al., 2001) and may well explain why the small neurons express MAP2. The expression of protein markers for both neurons and glia in small “neurons” or cells they generate suggest that they are probably a heterogeneous population. We noticed that in old embryonic cultures (DIV21), the number of small neurons did not greatly drop compared with that of younger cultures (DIV10 or DIV14), suggesting that not all the cells grow to generate astrocytes in the culture. The small neurons keep dividing, and some of them differentiate into astrocytes, but a proportion remain undifferentiated. Indeed, in DIV21 culture, we detected not many but a few NG2^+^/nestin^+^ cells that also express β-III tubulin (Fig. 7), but they seem not fully differentiated. Probably extra neurotrophic factors are needed for them to become mature neurons. In a word, the identity of these cells is complicated, and their fate is perhaps dependent on the extracellular environment.

In summary, we have identified a population of neuron-like precursor cells in mouse embryonic cortical cultures that express MAP2 but also nestin, Olig2, and NG2. During the time in culture, they are highly proliferative but steadily differentiate, mostly into GFAP^+^ astrocytes. True cortical neurons are present in the dish, but they are postmitotic and do not incorporate BrdU. We submit that these neuron-like precursors have been mistaken for cortical neurons in previous reports investigating neuronal cell cycle events *in vitro*. These studies would bear re-examining and the data surrounding the measurement of ectopic neuronal cell cycles should be reinterpreted if the neuron-like precursor cells were targeted.

## Synthesis

Reviewing Editor: Deanna Smith, University of South Carolina Decisions are customarily a result of the Reviewing Editor and the peer reviewers coming together and discussing their recommendations until a consensus is reached. When revisions are invited, a fact-based synthesis statement explaining their decision and outlining what is needed to prepare a revision will be listed below. The following reviewer(s) agreed to reveal their identity: Weikang Cai.

Both reviewers felt that this work is important, given the paucity of data regarding the identity of cell types in neuronal cultures. The finding that the small cells that are positive for both MAP2 and cell cycle markers are probably a glial progenitor is interesting and useful, as is the demonstration that this population, previously thought to be neuronal, is unaffected by Aβ-stimulated THP1 cell media treatment. The main concern is that the data supporting the hypothesis that this population consists of astrocyte precursors rather than other progenitors is not as strong as the title suggests. It would be useful to know whether the GFAP+ cells are glial precursors or mature astrocytes; better yet would be to demonstrate that it is actually the small cells that differentiate into astrocytes. This is admittedly difficult and would require substantial new experimentation. Reviewers felt that deemphasizing this in the title and text would be sufficient. The reviewers had some additional concerns which are included below.

Reviewer 1:

In this manuscript Zhang et al examine the identity of MAP2 positive (MAP2+) neurons that express cell cycle-related proteins in embryonic mouse cortical cultures. The authors find that these cells have smaller soma size than MAP2+ neurons that don’t express cell cycle markers (thereafter named “small neurons”). The authors then show that upon treatment of cultures with a toxic Aβ-conditioned THP1 cell media, the large soma sized neurons die, while the number of “small neurons” does not change. Further analysis into the identity of these “small neurons” shows that they express a neuronal precursor marker protein nestin, as well as glial lineage markers NG2 and Olig2, thus, even though they express MAP2, they are probably not mature post mitotic neurons. Authors speculate, these “small neurons” differentiate into astrocytes. Based on these findings, the authors propose a reevaluation of the conclusions obtained in previous studies regarding the mechanism of Aβ-mediated neuronal death (via cell cycle entry).

The author’s findings may have important implications on future studies that use embryonic cultures to investigate mechanisms of neuronal apoptosis. However, the lack of quantification and statistical analysis, as well as lack of some controls throughout the manuscript, raises concerns about the significance of the data and its interpretation, as detailed in the points below:

Major points:

1. The statement that the “small neurons” are giving rise to astrocytes is weakly supported, relying solely on qualitative assessment of observations summarized in Figs 4-5. Although authors did not detect mature oligodendrocyte markers in their cultures, they did not look beyond DIV14, a relatively short culture time. Importantly, authors did not perform any additional analysis to confirm the presence of mature, or maturing astrocytes, using known astrocyte markers, other than GFAP, which is also a well-known marker of radial glia progenitors.

Still, even if presence of “mature” glia cells is detected in the cultures, it will not be direct evidence they arose from the “small neurons” the authors describe. Drawing that conclusion will require rigorous analysis using lineage tracing and related methods, which may be beyond the scope of this study.

Thus, the title of the manuscript is misleading, as it implies discovery of a novel type of progenitor cells that give rise to astrocytes. The authors should either provide strong experimental evidence to support this statement, or change the title to one that better reflects the main findings of the study (e.g. that the “small neurons” are progenitors expressing glial lineage markers that have been mistaken for neurons, and are not susceptible to toxic perturbation by Aβ stimulated THP1-conditioned media, calling to reexamine previous findings using this paradigm).

2. Throughout the manuscript’s figures, the conclusions are made based on presence or absence of signal obtained by immunofluorescence experiments using antibody staining. The text describes “weak", “strong” or “diffuse” signal; however, no quantification of signal intensity or any similar measurement is presented to corroborate these descriptions. Authors should provide signal intensity quantification in every figure where such comparison is made (for example, line 235 related to Fig1-2: “..in experiments such as these were small in size with weak MAP2 staining..”, or, line 321.. related to Fig5 ..”exhibited weak diffuse NG2 signal..”).

Importantly, many antibody staining experiments can result in background signal which can be misinterpreted as a “weak” real signal. A control experiment should be performed and presented, where primary antibody is omitted, and only secondary antibody is applied, to determine the level of background.

3. Throughout the manuscript authors use text such as ..”significantly different from..” without any statistical analysis to base that on. (For example line 329-330 ..”the percentage of Olig2+ cells that were GFAP+ also significantly increased..). For each figure, authors should provide statistical analysis where it is appropriate. For example, statistical analysis is missing from Fig 1L, Fig4D, Fig5F-G.

In Fig2 C-D statistical analysis was done by t-test, however, since multiple groups are compared in the same experiment, an ANOVA analysis is more appropriate.

Fig3, and Fig6A-B - quantification of number of cells counted for each marker as stated in the text, along with the appropriate statistical analysis should be performed.

4. The Methods section lacks details about many procedures and reagents. The following should be provided:

• strain of animals used, animal housing, welfare, anesthesia method for perfusion.

• the amount of Aβ-treated THP1 media used to treat cortical cultures.

• details on vendors and concentrations of all reagents (only very few are listed).

• clear definition of small and big soma size neurons (range of sizes for each group).

• in each figure where cells were counted, provide number of cells counted for each marker, number of coverslips, and number of biological replicates (separate culture preps).

• detailed explanation on how imaging was done, e.g. magnification used, resolution, whether it was single plane of z stack.

• for images of the cortex in vivo (Fig 6C-E), what region of the cortex was imaged (visual, motor)? What area of the cortex (grey matter, deep layers etc).

5. In Fig2 authors show that number of “small neurons” is unaltered by the toxic media treatment. However, in previous study by the group, authors showed that cell cycle marker positive cells (named here “small neurons”) are dying, as shown by TUNEL staining (Wu et al 2000)). How does the present finding reconcile with previous work?

6. Fig6 C-E - add arrows in the MAP2 panel that correspond to cells stained with proteins as labeled in the adjacent panels. Are these cells expressing MAP2? What is the percentage of these cells out of total cells? In the text, there is reference to Supplementary figure (line 357, 359) - however this figure wasn’t submitted with the manuscript.

7. It was shown in Fig1 that CyclinD1 labels both small and large soma size cells (Fig1G, M), thus suggesting that mature postmitotic neurons can enter cell cycle under normal culture conditions. If the experiment described in Fig2 was repeated with CyclinD1 as marker, instead of EdU, and large soma CyclinD1 positive cells were analyzed, would there be increase in numbers following treatment? Were there any large soma cells positive for any cell cycle marker at early time points after the toxic media treatment (before the 24hr time point)?

Minor points:

1. A typo in the Methods section (line 183) - should be Fiji software?

2. Fig4D -error bars are missing

3. Fig6A-B - the MAP2 panel appears to have background from other channels, the quality of immunostaining or imaging in the presented experiment should be assessed.

Reviewer 2:

The manuscript entitled “Identifying a population of neuron-like progenitor cells that generate astrocytes in mouse cortical culture” aimed to address a critical question regarding the cellular identity and fate of the in vitro cultured primary cortical neurons. The authors used immunostaining to clearly show the heterogeneous nature of the cultured primary neurons, which may include mature neurons, immature neurons, neural progenitor cells, as well as some potential glial cell types. Both the neural progenitor cells and glial cells, such as astrocytes, are highly proliferative. The authors have provided convincing analysis demonstrating these proliferating neural progenitor cells are resistant to cellular death, while the non-proliferative neuron-committed cells in the culture are susceptible to apoptosis following neuroinflammatory stimulus. Together, these results raise a concern of the current methods to using in vitro cultured neurons to investigate the cell-cycle-related death. The present study provides a more comprehensive and rigorous analysis on primary cultured neurons due to its high degree of heterogeneity.

In general, the manuscript is clearly written and easy to understand. However, there are several concerns to be addressed.

1. The authors claimed that GFAP+/Nestin+ cells are astrocytes only based on the expression of GFAP. This is not sufficient and convincing. A population of neural progenitor cells are “radial glia” with high GFAP expression in the brain. The GFAP+/Nestin+/Olig2+ cells identified in the study may well be the neural progenitor cells, but not the differentiated astrocytes. Radial glia-like neural progenitor cells can proliferate. They can also explain the time-dependent increase in the population as shown in Fig. 5F,G. There’s no additional examination to further characterize whether they are truly functional astrocytes.

2. Throughout the manuscript, the authors used “smaller neurons” to differentiate from the “bigger” “true” neurons. This is very vague. These two major types of cells should be systemically and unbiasedly characterized. How are the “small” vs “big” neurons unbiasedly classified? It is mentioned “100um2” in size as a cut-off in the discussion. This should be made clear and precise in the “results” session as well. In addition, other morphological analyses on these different cells should be systemically performed and presented. For example, branch numbers, length of processes, etc.

3. It is also quite convincing to claim EdU+ cells as proliferating cells. DNA repair can also lead to EdU incorporation. This should be at least acknowledged.

4. Why is the percentage of the EdU+ cells smaller than those of cells with Ki67+, CyclinD1+, etc (Fig.1K)? Edu labeling should cover more proliferating cells as a tracing method, while staining of cell-cycle specific markers should only provide a “snapshot” of the total population and cover a less percentage.

Some of the data description and presentation are confusing and difficult to understand. These areas need to be edited to improve clarity.

1. Figure 2C,D may not be the good way to present. They are a bit hard to understand. An alternative presentation should be considered (e.g. stacked bars or pie chart). In Figure 2D, it is not clear that none “Big” Edu+ cells are present, although the manuscript describes so (much later in line 397). The relative changes in the figure and text should be made to improve the clarity. The discussion of the reason for an increase in percentage of the EdU+/MAP2+ neurons (line 409 in the Discussion) is better to be moved to the results (for example, to line 267) to guide the audience through the results.

2. In figure 5, while the “small” and “big” cells are pointed by arrows and arrowheads, and described in the figure legend, it is still better to label on the images to make it easier to read. In most cases, the top panels contain small cells, while the bottom panels contain big cells.

3. Some labeling of the figures should be clearer. For instance, Figure 2C, MAP2 count of what area? Figure 2D, what is the y axis presenting, fraction or percentage? Similar issues in Figure 5F and G.

4. Figure legends of Fig2 is problematic. EdU does not only label cells in the S phase. It labels cells with active DNA synthesis/repair or recently underwent DNA synthesis/repair. There are no panels E-H in Figure 2.
